# Birth order and sickness absence: register-based evidence from Finland

**DOI:** 10.1093/eurpub/ckac131.199

**Published:** 2022-10-25

**Authors:** K Reini

**Affiliations:** Åbo Akademi University, Vasa, Finland

## Abstract

**Background:**

In working ages, sickness absence is strongly related to persons’ health condition. We studied how birth order was associated with receipt of sickness absence, distinguishing mental disorders, musculoskeletal disorders and injuries.

**Methods:**

A follow-up study based on the entire Finnish population was conducted for sibling groups born 1969-1982, in which each sibling was observed from age 35 years in the period 2004-2018. Focus was on within-family variation in first-time sickness allowance receipt. Stratified Cox regressions were estimated using Stata 16.

**Results:**

Each increase in birth order was associated with a higher risk of sickness absence. For mental disorders, the hazard ratio as compared to first borns was 1.03 (95% CI: 0.98, 1.08) of second borns, 1.10 (95% CI: 0.99, 1.22) of third borns, and 1.52 (95% CI: 1.25, 1.85) of fourth or higher borns. Corresponding numbers for musculoskeletal disorders were 1.12 (95% CI: 1.07, 1.17), 1.19 (95% CI: 1.09, 1.30) and 1.15 (95% CI: 0.96, 1.38), and for injuries 1.06 (95% CI: 1.01, 1.12), 1.09 (95% CI: 1.21, 1.14) and 0.96 (95% CI: 0.77, 1.20), respectively. These associations were notably stronger for women than men, but modestly influenced by mother’s age at birth, educational level, occupation, income and family composition.

**Conclusions:**

The study was the first to provide evidence about an interrelation between birth order and sickness absence, utilising diagnose information and family fixed effects methods. A clear birth order pattern was observed for these main causes, but not for a residual group that consisted of other miscellaneous causes. Higher birth order was found associated with a higher risk of sickness absence, and particularly so for mental disorders and muscoloskeletal disorders in women. The underlying mechanisms behind these associations constitute an avenue for future research.

**Key messages:**

• Using sibling fixed effects, the study was the first to analyse if sickness absence by main cause is associated with birth order in working ages.

• First-born siblings were the least likely to be sick because of mental or musculoskeletal disorders, and the associations were notably more accentuated for women than men.

